# Multi-Faceted Roles of DNAJB Protein in Cancer Metastasis and Clinical Implications

**DOI:** 10.3390/ijms232314970

**Published:** 2022-11-29

**Authors:** Hye-Youn Kim, Suntaek Hong

**Affiliations:** 1 Laboratory of Cancer Cell Biology, Department of Biochemistry, Gachon University School of Medicine, 155 Gaetbel-ro Yeonsu-gu, Incheon 21999, Republic of Korea; 2 Department of Health Sciences and Technology, Gachon Advanced Institute for Health Sciences & Technology, Gachon University, Incheon 21999, Republic of Korea

**Keywords:** cancer metastasis, molecular chaperone, heat shock protein 40, DNAJB, cancer therapy

## Abstract

Heat shock proteins (HSPs) are highly conserved molecular chaperones with diverse cellular activities, including protein folding, assembly or disassembly of protein complexes, and maturation process under diverse stress conditions. HSPs also play essential roles in tumorigenesis, metastasis, and therapeutic resistance across cancers. Among them, HSP40s are widely accepted as regulators of HSP70/HSP90 chaperones and an accumulating number of biological functions as molecular chaperones dependent or independent of either of these chaperones. Despite large numbers of HSP40s, little is known about their physiologic roles, specifically in cancer progression. This article summarizes the multi-faceted role of DNAJB proteins as one subclass of the HSP40 family in cancer development and metastasis. Regulation and deregulation of DNAJB proteins at transcriptional, post-transcriptional, and post-translational levels contribute to tumor progression, particularly cancer metastasis. Furthermore, understanding differences in function and regulating mechanism between DNAJB proteins offers a new perspective on tumorigenesis and metastasis to improve therapeutic opportunities for malignant diseases.

## 1. Introduction

Heat shock proteins (HSPs) are a large family of molecular chaperones whose expression levels are increased in response to cellular stress, such as elevated temperatures and oxygen deprivation [1]. However, several HSPs are also present in cells under completely normal conditions. They are not responsive to heat stress. Instead, they are controlled by different stress signals [2,3]. HSPs are involved in numerous biological processes related to protein stability, including protein folding, unfolding, synthesis, stabilization, and degradation, thus maintaining cellular homeostasis and protecting cells from endogenous and environmental stresses, such as hypoxia, infection, heat shock, chemicals, and radiation [3,4]. HSPs can also regulate normal cell physiology in immunologic processes, cell cycle regulation, transcriptional activation, and signal transduction [5,6]. In mammals, HSPs are classified into six families based on their relative molecular weights, including small HSPs, HSP40, HSP60, HSP70, HSP90, and HSP100 [7,8]. Most HSPs are highly conserved, implying that they have essential roles in the physiology of mammalian cells. For example, HSP70 and HSP90 play central roles in controlling cell proliferation, apoptosis, and cell cycles, regulating the folding/refolding of various proteins for stabilization or degradation [6,9,10,11,12].

Among HSPs, HSP40s function as co-chaperones to regulate the activities of HSP70s by interacting with HSP70s and stimulating ATP hydrolysis with client proteins [13,14]. HSP40 proteins are also known as J-domain proteins because they contain the most conserved amino acid region called the “J-domain”, first identified in Escherichia coli DnaJ protein. Especially the universally conserved HPD motif (His33-Pro34-Asp35) in HSP40s is crucial for stimulating the ATPase activity of HSP70 [15].

**Figure 1:**
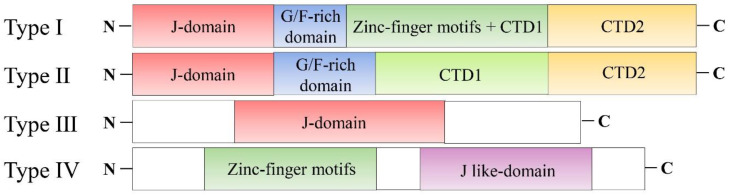
Organization and structures of four types of HSP40 proteins. HSP40s are classified into four subclasses (Type I, Type II, Type III, and Type IV). Type I class contains a J-domain, a glycine and phenylalanine-rich domain (G/F-domain), conserved zinc finger motif, and a client-binding domain (CTD). Type II class includes the J-domain, G/F-domains, and CTD, similar to Type I class, but lacks the zinc-finger domain. The Type III class only has a J-domain and lacks both G/F and zinc-finger domains. In contrast, Type IV class contains a J-like domain and conserved zinc finger motif.

HSP40s are the largest and most diverse protein families among HSPs, consisting of over 50 members. HSP40 families are further categorized into four subclasses according to their domain structures: Type I, Type II, Type III, and Type IV (Figure 1). Type I DNAJA contains an N-terminal J-domain, a glycine- and phenylalanine-rich domain (G/F), a zinc finger motif with conserved CXXCXGXG residues, and a C-terminal client-binding domain (CTD). Type II DNAJB includes an N-terminal J-domain, G/F-domains, and a CTD, similar to DNAJA class, but lacks the zinc-finger domain [13]. These Type I and Type II HSP40 subclasses function independently of ATP. They can bind to aberrant polypeptides to reduce cellular stress by preventing aggregation. The major difference between Type I and II HSP40s is that Type I DNAJA could function independently of HSP70, whereas Type II DNAJB must be associated with HSP70 to prevent aggregation of client proteins [16]. Type II DNAJB family is divided into three major subfamilies on the basis of their closest amino acid homology. The first group consists of the four members, such as DNAJB2, DNAJB6, DNAJB7, and DNAJB8. The second subfamily contains the DNAJB1, DNAJB4, DNAJB5, DNAJB9, DNAJB11 and DNAJB13 proteins. The last group consists of the members DNAJB12 and DNAJB14 [17].

Type III DNAJC only has a C-terminal J-domain. It lacks both G/F and zinc-finger domains. In contrast, Type IV DNAJD, a group of recently identified proteins classified as J-like proteins, lacks some essential residues. However, Type III and IV proteins have a similar J-domain structure and can function independently of HSP70 [18]. These HSP40 proteins can recognize their specific substrate through polypeptide binding domains and transport them to specific HSP70 members [16,19]. Unsurprisingly, accumulating evidence has indicated that HSP40s have a multi-faceted role in tumor progression and metastasis. In this review, we summarize Type II HSP40 subclass DNAJB proteins in detail to understand cellular functions and/or molecular mechanisms regulating tumor progression and metastasis. Furthermore, we describe future directions and challenges of using HSP40 subclass DNAJB proteins as therapeutic targets, which may help us design novel and appropriate targeted cancer therapies.

## 2. Regulation of DNAJB Proteins at RNA Level 

### 2.1. Transcriptional Regulation of DNAJB 

Although little has been studied regarding the regulation of DNAJB4 at the transcription level, some results have shown that there are putative transcriptional binding sites for DNAJB4. Promoter activity analysis showed that four YY1 binding sites were located in the DNAJB4 promoter within −232 and −122. YY1 regulates the expression of numerous genes primarily involved in tumorigenesis as a transcription factor. Overexpression of YY1 can induce DNAJB4 transcription by directly binding to the promoter region, which inhibits the invasive ability of non-small-cell lung carcinoma (NSCLC) cells [20]. Hepatitis B virus (HBV), the leading cause of human hepatocellular carcinoma (HCC), could also promote DNAJB4 transcription in HCC cells by upregulating YY1 [21]. HBV can promote metastasis-related genes and lead to metastasis of HCC cells [22,23,24]. However, specific functions of DNAJB4 in the progression of HCC metastasis remain largely unknown. 

AP-1 is a dimeric transcription factor complex that comprises members of the FOS, JUN, ATF, and MAF protein families. AP-1 proteins such as Fos and Jun are generally considered proto-oncogenes. However, some AP-1 proteins, such as JunB and c-Fos, can also suppress tumorigeneses [25,26,27]. Through further investigation, the AP-1 binding site (−1457 to −1451) has been identified in the DNAJB4 promoter, through which AP-1 can positively regulate DNAJB4 transcription [28]. Combined expression of YY1 and AP-1 can enhance DNAJB4 expression and suppress cancer cell proliferation, angiogenesis, and metastasis. Curcumin (diferuloylmethane), the most active component of turmeric, can induce DNAJB4 through the activation of JunD as a composition of the AP-1 transcription factor complex and suppress the invasion and metastasis of lung cancer cells [29]. 

Lens-specific transcription factor FOXE3 is indispensable for the early step of eye development and the formation of lens placode. Interestingly, combined transcriptome and proteome analysis has revealed that DNAJB1 has a downstream target of FOXE3, which plays a crucial role in developing and maintaining lens transparency [30]. 

DNAJB8 is a highly conserved testis-enriched gene, especially in postmeiotic germ cells. It functions as a cancer/testis antigen, causing tumorigenesis initiation [31]. Recently, it has been reported that transcription factor SOX30 can control the transcriptional activation of the DNAJB8 gene in mouse testis during late meiosis and spermiogenesis [32]. Although DNAJB8 is an evolutionally conserved testis-enriched gene, DNAJB8 knockout mice did not show functional defects in germ cell development or male fertility, suggesting that multiple redundant pathways might regulate spermatogenesis [32]. According to the above results, DNAJB families are implicated in diverse cellular functions in responses to various transcription factors. 

### 2.2. Post-Transcriptional Regulation of DNAJB 

MicroRNAs (miRNAs) are small non-coding RNAs that regulate gene expression by binding to target mRNAs. Numerous studies have demonstrated significant associations between miRNAs and various disease states. However, the emerging roles of miRNAs in regulating HSP40s, specifically DNAJB proteins, have not been clarified yet. The function of miR-623 has been limited in cancer progression. Specific target genes for miR-623 have not been reported yet. Mitra et al. have demonstrated that the DNAJB6 level is negatively regulated by miR-632 through knowledge-based screening [33]. An inverse correlation between DNAJB6 mRNA level and miR-632 expression has been observed in breast tumor specimens. Overexpression of miR-632 is significantly linked to reduced DNAJB6 protein expression, promoting invasiveness of breast cancer cells concomitant with increased expression of mesenchymal proteins such as ZEB2 and Slug and reduced expression of E-cadherin. These findings suggest that miR-632 and DNAJB6 can be important biomarkers for metastatic and malignant progression of breast cancer and that targeting miR-632 may provide novel strategies for inhibiting cancer metastasis [33]. 

DNAJB1 plays a key role in assuring the quality of proteins as a chaperone or co-chaperone. It is known to be involved in the inherited neuropathogenesis of polyglutamine (poly Q) diseases such as Spinocerebellar ataxia type 3 (SCA3) [34]. In SCA3 patient-derived induced pluripotent stem cell line, miR-370 and miR-543 expression levels are significantly upregulated, while the expression of DNAJB1 is downregulated, suggesting that both miR-370 and miR-543 can negatively cooperate to suppress the transcription of DNAJB1. This result indicates that aberrant expression of miR-370 and miR-543 is related to the pathogenesis of SCA3. These miRNAs are also implicated as novel therapeutic targets and/or diagnostic markers [35]. 

DNAJB9 has been recently identified as the target gene of miR-32 in acute myeloid leukemia (AML) [36]. DNAJB9 expression is inversely correlated with miR-32 and positively correlated with small nucleolar RNA host gene 5 (SNHG5) in AML cells. SNHG5 is a long non-coding RNA that can bind to specific target miRNAs for sequestering, ultimately upregulating miRNA target gene expression [36]. Thus, SNHG5 could function as a miR-32 sponge to abolish miR-32-induced repressing activity on DNAJB9. Creating a complex between SNHG5/miR-32/DNAJB9 possibly causes chemotherapy resistance in AML cells [37]. The comparable regulatory network has also reported that miR-152-3p can negatively regulate DNAJB12, while lncRNA HCG18 can promote DNAJB12 by competitively binding to miR-152-3p, which enhances gastric cancer cells proliferation, migration, and invasion [38].

## 3. Post-Translational Regulation of DNAJB Proteins

Post-translational modifications (PTMs) can be categorized into four groups: (1) addition of polypeptide chain involving ubiquitin (ubiquitination), SUMO (SUMOylation), ISG (ISGylation), and NEDD (NEDDylation), (2) addition of a small chemical/ionic group (an acetyl, methyl, and phosphate group), (3) addition of carbohydrate molecules (glycosylation, ADP ribosylation), and (4) lipid molecule-based modifications (palmitoylation, prenylation, myristoylation). They often occur in response to cellular environment changes [39]. By modifying proteins in these ways, PTMs are involved in critical biological processes, including gene expression regulation, DNA repair, and cell signal transduction [40,41]. However, aberrant PTMs can cause several human pathologies, including neurological diseases and cancers [42]. Accumulated evidence suggests that HSPs are highly modified at the post-translational level in multiple ways, particularly through phosphorylation, methylation, acetylation, AMPylation, NEDDylation, and ubiquitination [43,44]. 

### 3.1. Phosphorylation of DNAJB Protein

DNAJB1 can be phosphorylated by protein kinase CK2 at four sites in its cytosolic domain [45]. CK2 actually phosphorylates DNAJB1 through direct interaction. Therefore, phosphorylated DNAJB1 might translocate from the cytosol to the nucleus. Epigenetic modification of protein phosphorylation is a well-known mechanism to control nuclear import/export by many kinds of kinases and phosphatases [46]. 

Another study has shown that DNAJB1 is a substrate for mitogen-activated protein kinase-activated protein kinase 5 (MK5) [47]. MK5 can phosphorylate DNAJB1 at Ser-149, Ser-151, and Ser-171 of its C-terminal domain. Functionally, MK5-stimulated DNAJB1 phosphorylation can facilitate DNAJB1-mediated repression of the transcription activity of heat shock factor 1. In this regard, drug development targeting MK5 kinase might show therapeutic benefits for cancer patients [47]. 

Although the role of nuclear-translocated DNAJB1 by phosphorylation was not clear, the previous computational analysis suggested that the cytosolic domain of DNAJB1 contains two putative DNA binding domains [48,49]. Released cytosolic domain after phosphorylation translocated into the nucleus and may work as transcription factor to regulate gene expression at the transcriptional level. Further studies are required to understand the exact role of phosphorylated DNAJB1 by protein kinases.

### 3.2. Glycosylation of DNAJB Protein 

Glycosylation is the most important and frequent post-translational modification that occurs mainly in the cytosol, endoplasmic reticulum (ER), and Golgi apparatus [50]. Glycosylation includes N-linked, O- linked, and C-linked glycosylation, depending on the type of glycosidic linkage. This PTM is critical for a wide range of biological processes, such as protein-protein interactions and cell attachment to the extracellular matrix in cells. Deregulation of protein glycosylation can cause various human diseases such as Alzheimer’s disease, cancer, inflammation, and diabetes [51,52,53]. 

DNAJB11 (also known as HEDJ) not only regulates the folding of misfolded or unfolded proteins but also exports unfolded proteins to the cytosol for proteasomal degradation. 

N-linked glycosylation of DNAJB11 at two potential N-linked glycosylation sites has been confirmed through EndoH and PNGase glycosidase treatment [54]. DNAJB11 glycosylation induces endoglycanase and reverses translocation into the ER and luminally orient. However, the retrograde transport mechanism has not been elucidated yet [54]. Elucidation of these underlying mechanisms is important because aggregated or misfolded proteins in the ER can cause ER stress, which can trigger multiple human diseases such as neurodegenerative disorders [55]. Collectively, investigating the role of DNAJB11 in the folding and translocation of misfolded proteins and performing a mechanistic study of DNAJB11 glycosylation might provide insight into cellular biology and the pathogenesis of diverse human diseases [54].

### 3.3. Acetylation of DNAJB Protein

Conformational protein changes and maintaining protein folding in living cells are major challenges in the cellular environment. Misfolding and aggregation of distinct proteins are associated with various human diseases, including Parkinson’s disease, Alzheimer’s disease, type 2 diabetes, and familial lateral sclerosis [56,57,58]. Additionally, polyglutamine (poly Q) diseases such as Huntington’s disease are age-related neurodegenerative diseases caused by polyQ protein with an expanded poly Q stretch that can misfold, aggregate, and subsequently accumulate as inclusion bodies within neurons [59]. Cells possess a complex chaperoning network to prevent these pathological protein aggregations and promote efficient protein folding [60]. DNAJB6b and DNAJB8 of the HSP40 subclass have been identified as strong poly Q aggregation inhibitors in a way that is dependent on HDAC4-induced DNAJB6b and DNAJB8 deacetylation on lysine K216 and K223. Therefore, the functional relationship between HDAC4 activity and (de)acetylation of DNAJB6b and DNAJB8 offers a fascinating approach to treating aging-related protein folding diseases [61].

## 4. Multi-Faceted Roles of DNAJB Protein in Cancer Metastasis

**Figure 2:**
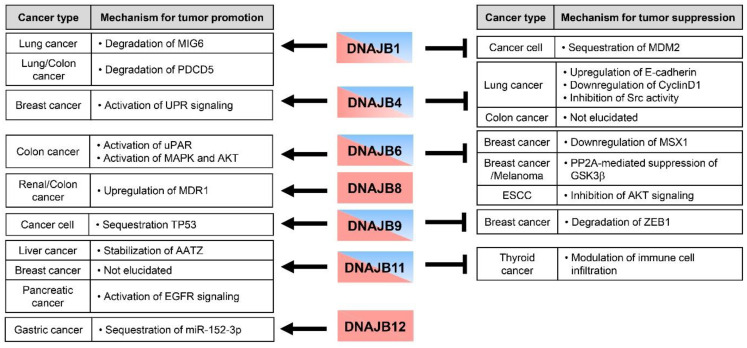
Multi-faceted roles of DNAJB protein in cancer pathology. DNAJB proteins act both as tumor promoter (red color) or suppressor (blue color) depending on tissue type and context-dependent manner. DNAJB8 and JB12 proteins promote tumor growth and metastasis in several cancers. Interestingly, other DNAJB proteins have divergent roles in cancer development and metastasis as promoter or inhibitor.

Metastasis and invasion as hallmarks of cancerous cells require several sequential cellular processes, including cancer cells leaving their local site and intravasation into the bloodstream, enduring blood pressure in circulation, and extravasation to lode and proliferating successfully in new secondary organs [62]. These different stages are related to various molecular switches that regulate cell proliferation, angiogenesis, migration, and invasion into surrounding tissues or organs. Although the landscape of investigation intended for reducing tumor growth and anticancer drug resistance has been developed, metastasis is still the primary cause of cancer death [63]. Many studies have revealed many potential roles of HSP40 subclass DNAJB proteins in cancer invasion and metastasis by regulating multiple signaling pathways (Figure 2) [64].

### 4.1. DNAJB1/HDJ1

**Figure 3:**
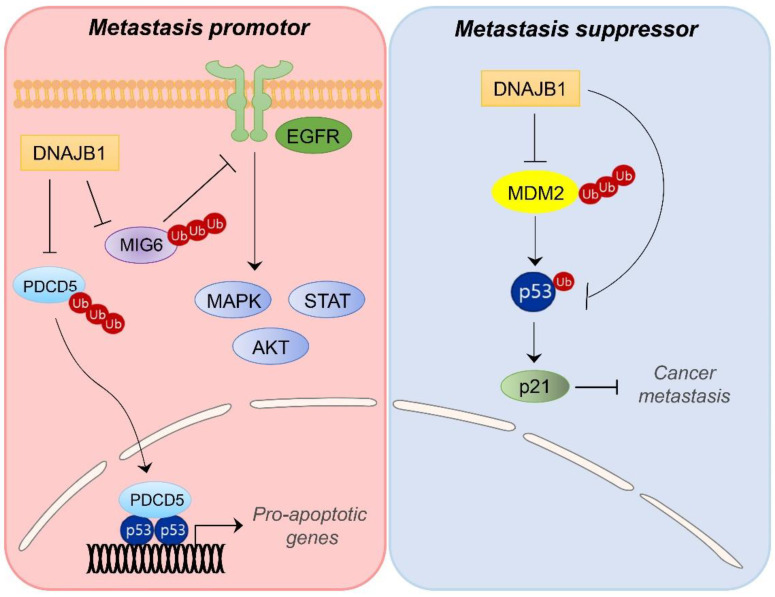
Schematic model showing divergent roles of DNAJB1 in cancer metastasis. DNAJB1 can play as a metastasis promoter or suppressor, depending on cancer cell type and context-dependent manner.

DNAJB1 has been shown to be associated with various cellular processes, including the proteasome pathway, ER stress, and viral infection [65,66,67]. Accumulating reports have indicated that DNAJB1 is an unfavorable prognostic marker in cancer progression and therapeutic resistance [68,69,70]. In lung cancer, DNAJB1 is required for epidermal growth factor receptor (EGFR) signaling activation by inducing proteasomal degradation of mitogen-inducible gene 6 (MIG6), leading to enhanced proliferation of lung cancer cells (Figure 3) [69]. Additionally, DNAJB1 can degrade tumor suppressor PDCD5, a promotor of p53-mediated apoptosis, through ubiquitin-dependent proteasomal pathway. Negative regulation of PDCD5-induced apoptosis by DNAJB1 leads to enhanced proliferation of lung and colon cancer cells [71]. In cholangiocarcinoma (CCA) and pancreatic cancer, increased DNAJB1 expression is associated with pathologically advanced cancer, tumor stage, lymph node metastasis, and clinical stage in patients [72]. It has been suggested that DNAJB1 has obvious novel diagnostic and prognostic implications for CCA and pancreatic cancers as an unfavorable biomarker for patient survival [72].

In contrast, DNAJB1 also interacts with MDM2 to induce the accumulation of MDM2 at the post-translational level [73]. Then, DNAJB1 suppressed the MDM2-induced degradation of p53, thereby promoting p53-mediated apoptosis in cancer cells (Figure 3). Tumor-suppressive activity of DNAJB1 through MDM2-mediated p53 regulation needs further study to confirm in human cancers.

### 4.2. DNAJB4/HLJ1

DNAJB4 is implicated in skeletal muscle proteostasis [74] and Alzheimer’s disease [75]. In addition, accumulating studies have suggested that DNAJB4 is involved in various cancers as a tumor suppressor that could inhibit cancer cell proliferation, invasion, and metastasis. High levels of DNAJB4 are associated with better overall survival and disease-free survival rates in NSCLC patients [20]. As a mechanism, the YY1 transcriptional factor can positively regulate DNAJB4 expression, thus reducing lung cancer cell invasive ability by upregulating E-cadherin expression [20]. Moreover, DNAJB4 overexpression can inhibit the proliferation, tumorigenesis, cell mortality, and invasion of lung cancer cells by reducing cyclin D1 expression, increasing STAT1 and p21WAF1 expression, and then activating the STAT1 pathway [76]. Chen et al. have also shown that curcumin can induce DNAJB4 expression by activating the JNK/JunD signaling pathway and attenuating metastasis capabilities of lung cancer cells by increasing E-cadherin expression [29]. Additionally, suppressing the catalytic activity of Src by DNAJB4, which functions as an endogenous Src inhibitor, can downregulate EGFR, FAK, and STAT3 downstream signaling pathways, leading to inhibition of epithelial–mesenchymal transition (EMT) and metastasis of lung cancer cells [77]. 

DNAJB4 has also been recognized as a tumor suppressor in colorectal carcinoma. DNAJB4 expression is lower in highly metastatic colorectal cancer cells than in poorly metastatic ones [78]. Ectopic overexpression of DNAJB4 can dramatically inhibit colorectal cancer cell invasion and metastatic potential. Patients with higher DNAJB4 levels have better overall survival rates than those with lower DNAJB4 levels. DNAJB4 is a potent tumor suppressor of colorectal cancer (CRC). It has been suggested to be a predictive clinical marker for CRC patients [78]. 

However, recent proteomic and patient data analyses have shown that DNAJB4 is a potential metastasis promoter in breast cancer cells [79]. DNAJB4 mRNA level is significantly higher in mesenchymal cells than in epithelial breast cancer cells. In particular, the knockdown of DNAJB4 in highly metastatic MDA-MB-231 cells can decrease the migration and metastasis of breast cancer cells and suppress the primary tumor growth rate. Consistent with these observations, high levels of DNAJB4 are correlated with a poor metastasis-free survival rate in patients with breast cancer [79]. In support of this, activation of unfolded protein response signaling pathway in response to EMT under cellular stress may induce DNAJB4 expression as a chaperone protein [80]. Molecular mechanisms of DNAJB4 regulation during EMT require further investigations to improve clinical outcomes of patients with metastatic breast cancers. 

### 4.3. DNAJB6/MRJ

**Figure 4:**
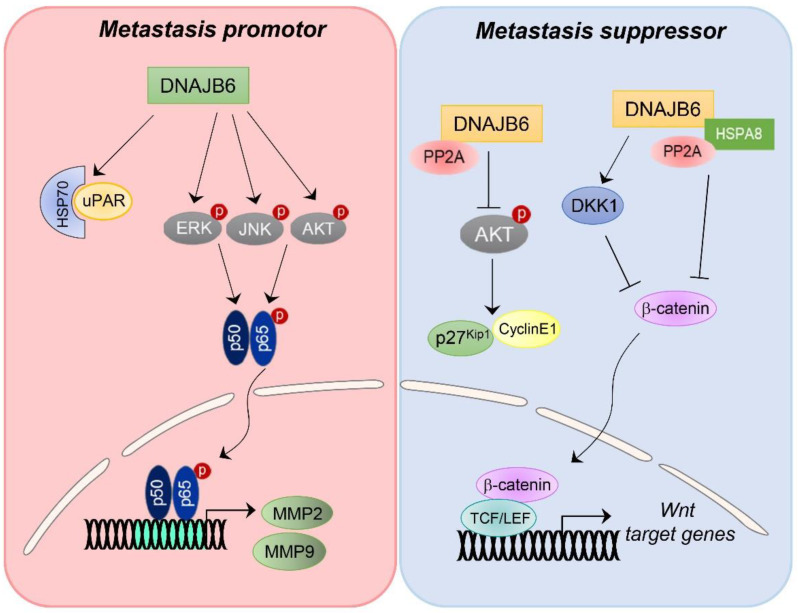
Schematic model showing multi-faceted roles of DNAJB6 in cancer metastasis. DNAJB6 can function as a metastasis promoter or suppressor depending on cancer cell type and context-dependent manner.

DNAJB6 proteins comprise two differentially alternative splicing variants, DNAJB6a (the longer nuclear isoform) and DNAJB6b (the shorter cytoplasmic isoform). Both DNAJB6 isoforms contain the conserved N-terminal J domain, glycine-phenylalanine-rich domain (G/F-rich) residues, and a serine-rich region located in the C-terminal. However, DNAJB6b has a truncated carboxyl terminus [81]. Accumulating studies have reported that DNAJB6 could regulate not only various cellular physiological events such as cell polarity, cell morphology, stabilization, and mediation of protein–protein interaction but also plays diverse roles in multiple pathologies such as degenerative nervous system disorders, dementia, inherited myopathy, infectious viral diseases, and cancer [81,82,83,84]. The Wnt/β-catenin signaling pathway is one of the critical cascades regulating cancer cell proliferation, EMT, and metastasis in numerous types of cancer. Previous studies have reported that DNAJB6 is involved in cancer cell proliferation, EMT, and metastasis by regulating the Wnt/β-catenin signaling pathway in various cancers (Figure 4) [85,86]. DNAJB6 can downregulate MSX1, a β-catenin downstream target gene that is a transcriptional repressor of DKK1 [85]. Because DKK1 is a well-known Wnt/β-catenin signaling inhibitor, DNAJB6 can suppress Wnt/β-catenin signaling by upregulating the expression of DKK1. Therefore, a novel regulatory loop of β-catenin, MSX1, and DKK1 involving DNAJB6 represents the aberrant expression of DNAJB6 causing tumor growth, EMT, and metastasis of breast cancer [85]. Parallelly, DNAJB6 overexpression can induce the degradation of β-catenin and suppress Wnt/β-catenin signaling in breast cancer and melanoma cells [87]. It has been further revealed that the DNAJB6-HSPA8 (HSP70) complex in PP2A-dependent dephosphorylation of GSK3β as a client protein can reduce the abundance of β-catenin and subsequent loss of TCF/LEF (T cell factor 1/lymphoid enhancer factor1) activity, leading to suppression of EMT, tumor growth, and metastasis potential in breast and melanoma cancers [87].

In concordance with the suppressive role of DNAJB6 in cancer metastasis, high nuclear DNAJB6 expression levels are inversely correlated with outcomes in esophageal squamous cell carcinoma (ESCC) patients [88]. Moreover, there is a close negative correlation between the nuclear level of DNAJB6 and the presence of lymph node metastasis. Their conserved J-domain is crucial for tumor suppressive effects by down-regulating AKT1 signaling. DNAJB6 knockdown in ESCC cells can promote their proliferation and lymph node metastasis. Taken together, nuclear DNAJB6 plays a critical role in the development and lymph node metastasis of ESCC, reducing AKT signaling and suggesting its potential for development as a prognostic and therapeutic biomarker for ESCC [88]. 

In contrast, high expression of DNAJB6 promotes the aggressiveness of colon cancer cells (Figure 4). HSP70 and DNAJB6 can form a complex with uPAR to promote the adhesion and migration of HCT116 cells [89]. Over-expression of DNAJB6 can enhance the interaction between HSP70 and uPAR, while knockdown of DNAJB6 suppresses uPAR expression in HCT116 cells, suggesting that DNAJB6 might act as a uPAR-specific adaptor protein that links uPAR to HSP70. In support of this, the knockdown of DNAJB6 can inhibit uPAR-mediated cell adhesion and suppress cell invasion and migration by inhibiting invasion-related genes, including MMP2 and MMP9. Furthermore, upregulation of DNAJB6 can enhance phosphorylation of ERK, JNK, and AKT that is correlated with the MAPK pathway, providing novel insight into DNAJB6 functions as an inducer of cell adhesion and migration and as a therapeutic target in metastatic colon cancer [89].

### 4.4. DNAJB8

DNAJB8 is well established as an effective immunotherapy target in cancer stem cells (CSCs) and cancer-initiating cells (CICs) due to its identified functions in the maintenance of CSCs and CICs of renal cell carcinoma [31]. Similarly, DNAJB8 is overexpressed in CSCs and CICs of human colorectal cancer [90]. Exogenous expression of DNAJB8 can enhance the tumorigenicity of CRC cells with increasing levels of stem cell markers, indicating that DNAJB8 is a promising therapeutic target for immunotherapy against CSCs and CICs in CRC patients [90]. In another study, the same group has elucidated that heat shock factor 1 can increase DNAJB8 and SOX2 expression and induce CSCs and CICs [91]. As a new regulatory mechanism of DNAJB8 in tumor progression, the functional importance of maintaining CSCs and CICs has been highlighted to develop novel therapeutic strategies.

DNAJB8 can also inhibit TP53 through ubiquitin degradation in human colon cancer by directly interacting and upregulating MDR1, leading to increased oxaliplatin resistance to colon cancer [92]. Small extracellular vesicle (sEV)-mediated transfer of DNAJB8 could induce the resistance to oxaliplatin of colon cancer cells. Additionally, high levels of DNAJB8 in both colon tissues and serum are correlated with worse overall survival of colon cancer patients. These findings support that DNAJB8 level in serum sEV may serve as a potential biomarker for colon cancer and might be a promising therapeutic target for oxaliplatin-resistant cancer. Although DNAJB8 facilitates tumor development and progression, whether DNAJB8 is involved in tumor metastasis remains unclear.

### 4.5. DNAJB9/MDG1

DNAJB9 molecular chaperone was uncovered recently, and its precise function is largely unknown. DNAJB9 is located within the ER, where it acts as a co-chaperone for GRP78-binding immunoglobulin protein, the ER member of the Hsp70 [93]. DNAJB9 is known to be regulated by ER stresses such as heat, ethanol, sodium chloride, tunicamycin, and thapsigargin [93,94,95,96]. A recent study has revealed that DNAJB9 plays a key role in controlling TP53-mediated apoptosis under genotoxic stress [97]. The J-domain of DNAJB9 can bind to TP53 to suppress TP53-mediated Ras/Raf/Erk pathway, representing a negative feedback loop [97]. Another study from the same group has shown that DNAJB9 can overcome TP53-dependent senescence by interacting with TP53 and promoting cellular transformation [98]. These results might suggest that DNAJB9 promotes tumorigenesis through suppression of TP53-mediated senescence and cell death. 

In a recent study, DNAJB9 functions as a cytosolic regulator and as a soluble ER luminal protein that can interact with ΔF508-CFTR (F508 deletion mutation of cystic fibrosis transmembrane-conductance regulator), which generally induces cystic fibrosis causing ΔF508-CFTR degradation through ER-associated degradation pathway. As a result, DNAJB9 could be used as a therapeutic target for cystic fibrosis [99]. Recently, our study has revealed novel functions of DNAJB9 as a metastasis suppressor in breast cancer [100]. Our preclinical models and clinical bioinformatics analyses have demonstrated that lower DNAJB9 expression in aggressive breast cancer than in normal breast tissues is linked to worse cancer patient outcomes. Moreover, overexpression of DNAJB9 in highly metastatic breast cancer cells showed a more spherical cell morphology, reduced expression levels of mesenchymal markers, and metastatic abilities. Mechanistically, DNAJB9 can interact with FBXO45 (F-box/SPRY domain-containing protein 1) ubiquitin ligase to promote FBXO45 protein stability, which reduces the abundance of ZEB1 by proteasomal degradation, leading to repressed EMT, invasion, and metastasis of breast cancer cells [100]. Our study provides evidence for the feasibility of DNAJB9 as a potential therapeutic target for preventing cancer metastasis and a candidate metastasis inhibitor in other cancer types.

### 4.6. DNAJB11/ERdj3

DNAJB11 is upregulated along with its misfolded or aggregated client proteins when HSP70 molecular chaperone becomes overwhelmed, engaging in combating protein misfolding that is missed by HSP70-mediated quality control in the ER lumen [101]. Additionally, secreted DNAJB11 can attach to misfolded extracellular proteins, thereby protecting cells against the cytotoxic effects of misfolded proteins accumulated around cells. Concerning cancer, DNAJB11 is endogenously overexpressed in multiple types of human carcinoma cell lines, including Huh7, SH-SY5Y, and HeLa [101]. Specifically, DNAJB11 co-localized with AATZ (Z mutant of alpha-1-antitrypsin) protein can induce cirrhosis, increase cancer therapy tolerance, inhibit post-transcriptional degradation of AATZ, and promote tumorigenic AATZ polymer formation. As a result, DNAJB11 can promote HCC cell proliferation, EMT, invasiveness, and in vivo tumor growth, depending on AATZ. Taken together, a promising therapeutic strategy for the AATZ degradation pathway targeting DNAJB11 might provide a useful tool for drug development against HCC [102,103]. In concordance with previous results, DNAJB11 expression is significantly upregulated in breast cancer cell lines than in normal cells [104]. High DNAJB11 expression is correlated with poor overall survival, relapse-free survival, and distant metastasis-free survival, implying that DNAJB11 can be used as a prognostic marker for breast cancer patients. The eukaryotic promoter database shows that the DNAJB11 promoter region is hypomethylated in breast cancer patients’ tissues. Therefore, DNAJB11 expression in breast tumors might be controlled through epigenetic mechanisms such as histone modifications and miRNA expression other than DNA methylation [104]. Additionally, exosomal DNAJB11 promoted the proliferation, migration, and invasion of pancreatic ductal adenocarcinoma through the activation of EGFR and downstream signaling pathway [105]. Mechanically, DNAJB11 interacts with HSPA5 protein to block the unfolded protein response (UPR)-mediated apoptosis of pancreatic cancer. 

In contrast, a low level of DNAJB11 mRNA is associated with poor prognosis in thyroid carcinoma [106]. Through bioinformatics analysis using The Cancer Genome Atlas (TCGA) database, DNAJB11 was identified as a tumor suppressor which is downregulated in thyroid cancer tissues compared to normal ones. Especially the level of DNAJB11 was inversely correlated with the tumor and metastasis stage of thyroid cancer. Although the exact tumor suppressive activity of DNAJB11 was not identified, modulation of immune cell infiltration might affect the tumor growth and metastasis of thyroid cancer.

### 4.7. DNAJB12

The expression of DNAJB12 was upregulated in gastric cancer cell lines than in normal gastric cells by suppressing miR-152-3p-mediated DNAJB12 mRNA degradation [38]. In this study, Long non-coding RNA, HCG18, promoted the proliferation, migration, and metastasis of gastric cancer cells in an HNF1A-dependent manner. Upregulated HCG18 enhanced the stability of the DNAJB12 gene by sequestering miR-152-3p as a miRNA sponge. As a result, high DNAJB12 promoted the tumor growth and metastasis of gastric cancer by increasing PCNA and Vimentin but decreasing E-cadherin expression. In another study, DNAJB12 also protects hepatoma cancer cells from ER stress-induced apoptosis [107]. Mechanically, DNAJB12 suppresses the accumulation of proapoptotic BOK protein and the processing of caspases to contribute to the resistance against chemotherapeutics in liver cancer.

## 5. Current Approaches and Challenges for Targeting DNAJB Protein in Cancer Therapy 

Despite advances in anticancer drug development against various targets over the last decade, the field of HSP40 is still in its infancy. Currently, there is no emerged candidate treatment for HSP40 in clinical trials or promising lead compound for clinical development. Due to the various diversity of HSP40s, it is likely to be challenging to identify a single molecular scaffold. Many studies have reported that specific inhibition of HSP40 is still limited. Because HSP40 functions by interacting with their substrate proteins or HSP70 chaperone, effective strategies of HSP40 targeting inhibitors are being explored to block the interaction between a substrate protein and HSP70. Although the exact underlying mechanism of how DNAJB protein is involved in cancer development and metastasis needs further investigation, the development of novel therapeutics or combining chemotherapeutic agents with other HSP40 inhibitors can be applied to show synergistic anticancer effects against DNAJB proteins [69,108,109,110,111,112,113,114].

For example, KNK437, a benzylidene lactam compound and a pan-HSP inhibitor exerts its anti-tumor activity by inhibiting the expression of HSPs such as HSP27, HSP40, HSP72, and HSP110 [108,109]. KNK437 can significantly suppress the level of DNAJA1, decrease cell proliferation in vitro, and inhibit tumor growth and metastasis of CRC cells in vivo [110]. BMS-690514, a novel pan-HER/VEGFR inhibitor, can sensitize erlotinib-resistant NSCLC cell lines by promoting proapoptotic and cell cycle inhibitory factors or suppressing anti-apoptotic and heat shock proteins (HSP40, HSP70, and HSP90) [111]. Phenoxy-N-arylacetamide has also been identified as a novel small molecule inhibitor that can disrupt HSP70/HSP40 complex by binding to HSP40 directly [112]. However, as a therapeutic tool against cancer, it is still in its initial stage of drug development. In another study, R115777, a farnesyltransferase inhibitor, can suppress the growth, survival, and angiogenesis of breast cancer by modulating the farnesylation of DNAJA1 [113]. Combinational therapy of R115777 with classical paclitaxel drug can enhance drug sensitivity in breast cancer by decreasing VEGF and MMP-1. R115777 can also promote radiosensitization of glioblastoma multiforme cells by modulating the translocation of DNAJA1 from the cytosol into the nucleus [114]. Although this inhibitor does not target DNAJB proteins, it could be used to find a modulator for the post-translational modification of DNAJB proteins. 

Because DNAJB1 can promote the growth of lung cancer by enhancing EGFR signaling through K48-linked ubiquitination of MIG6, modulation of DNAJB1 might provide a novel therapeutic approach. As a matter of fact, suppressing DNAJB1 can increase the cytotoxicity of EGFR inhibitors through the stabilization of MIG6 [69]. In the case of DNAJB8, it is involved in the development and drug resistance of renal cell carcinoma through the maintenance of CICs [115]. Because gene targeting of DNAJB8 can reverse chemoresistance against docetaxel of renal cell carcinoma cells, genome editing technologies such as ZEN, TALEN, and CRISPR/Cas9 might be valuable tools to develop novel strategies to treat chemoresistant cancers. DNAJB6 has also been revealed as an independent prognostic factor for CRC patients. Knockdown of DNAJB6 can inhibit the metastasis and invasion of CRC cells in both in vitro and in vivo models, concomitantly suppressing IQ-domain GTPase-activating protein 1 and ERK phosphorylation [116]. In limb-girdle muscular dystrophy type D1 (LGMDD1), DNAJB6 mutation within the G/F domain can lead to an impaired interaction between DNAJB6, Z-disc protein such as desmin, and HSP70 and eventually result in misfolded myofibrillar protein aggregation and vacuolar myopathology [117]. As a small-molecule inhibitor, JG132 can inhibit the DNAJB6-HSP70 complex and rescue HSP70 mobility and muscle phenotypes in LGMDD1 mice. Because these small-molecule inhibitors are expected to inhibit chaperone–co-chaperone interaction and are most effective exclusively, it may be challenging to find more selective inhibitors against various human cancers. 

Natural-derived Hsp40 inhibitors, including myricetin, andrographolide, quercetin, epicatechin gallate, and curcumin, can disrupt the interaction between HSP40 and HSP70. Myricetin can bind to an allosteric site of HSP70, leading to a change in the conformation of nucleotide-binding domain that can inhibit the HSP40 protein’s binding to HSP70 and stimulate ATP hydrolysis [118]. Andrographolide, another natural product isolated from a Chinese herb, can influence the function of HSP40 [119]. Via promoter activation screening, andrographolide has been identified to show potential to activate the DNAJB4 promoter. This natural product does not require direct interaction with DNAJB4. However, it can activate the transcription of DNAJB4 through AP-1 sites in the DNAJB4 promoter. Increased DNAJB4 by andrographolide can suppress the metastatic ability of NSCLC cells [20]. As another DNAJB4 activator compound, curcumin can also induce the expression of DNAJB4 by activating the promoter in an AP-1-dependent manner [29]. Curcumin can inhibit the migration, invasion, and metastasis of lung cancer cells by activating DNAJB4, similar to andrographolide.

Although natural compounds have shown anticancer activities by stimulating several HSP40 proteins, these compounds are not specific for DNAJB proteins. To overcome this limitation, the development of new therapies has been incessant. Recently, a proteolysis-targeting chimera (PROTAC), which selectively degrades a protein of interest, has been shown to be innovative and promising in the treatment of many diseases, including cancer [120,121,122]. Traditional inhibitors capable of inhibiting the HSP90, which induces stabilization and activation of oncoproteins, still have some limitations in their applications. However, HSP90-interacting small-molecule-based PROTAC strategy has been developed to improve the therapeutic effect. Notably, HSP90 inhibitor compound 16b (BP3)-based PROTAC can effectively degrade HSP90 and suppress breast cancer cell growth in both in vitro and in vivo models [123]. To date, Arvina’s ARV110 and ARV-471 as PROTAC molecules targeting androgen receptor and estrogen receptor, respectively, have been approved by FDA for the treatment of metastatic breast cancer. Taken together, although PROTAC targeting DNAJB protein has not been developed yet, the PROTAC field has evolved and extended in the last decade. Accumulating experimental evidence suggests that PROTACs have an incredible potential to become an innovative therapeutic strategy for treating metastatic cancers by targeting DNAJB proteins.

## 6. Conclusions

We provided an overview of the HSP40 subclass DNAJB proteins mainly by focusing on their multi-faceted functions in tumorigenesis and metastasis. Not surprisingly, increasing evidence points out the pivotal role of several DNAJB proteins in maintaining protein homeostasis in numerous diseases, including cancers. Up to date, specific functions of six DNAJB proteins (DNAJB1, JB4, JB6, JB8, JB9, and JB11) among twelve members in tumorigenesis and metastasis have been clarified. Other poorly understood DNAJB proteins might also have potential roles in carcinogenesis and/or other diseases. In the last decade, HSPs have been used as therapeutic targets for cancer treatment, including HSP40s, prompting the development of novel chemotherapeutic agents. To date, effective drugs targeting HSP40 proteins directly have not been developed yet. However, targeting critical signaling nodes in the HSP40s-driven tumor progression pathway provides us an opportunity for the development of novel anticancer drugs. In addition, the application of PROTACS, a rapidly emerging novel technology, could facilitate the development of more effective therapeutic strategies to improve the clinical outcomes of patients with metastatic cancers.

## Data Availability

Not applicable.

## Abbreviations

AATZ	Z mutant of alpha-1-antitrypsin
AML	Acute myeloid leukemia
CCA	Cholangiocarcinoma
CIC	Cancer-initiating cell
CRC	Colorectal cancer
CSC	Cancer stem cell
CTD	Client-binding domain
EGFR	Epidermal growth factor receptor
EMT	Epithelial–mesenchymal transition
ER	Endoplasmic reticulum
ESCC	Esophageal squamous cell carcinoma
FBXO45	F-box/SPRY domain-containing protein 1
HBV	Hepatitis B virus
HCC	Hepatocellular carcinoma
HSP	Heat shock protein
LGMDD1	Limb-girdle muscular dystrophy type D1
MIG6	Mitogen-inducible gene 6
miRNA	MicroRNA
MK5	Mitogen-activated protein kinase-activated protein kinase 5
NSCLC	Non-small-cell lung carcinoma
PROTAC	Proteolysis-targeting chimera
PTM	Post-translational modification
sEV	Small extracellular vesicle
SNHG5	Small nucleolar RNA host gene 5
TCGA	The Cancer Genome Atlas
UPR	Unfolded protein response
